# Plasma-Derived Exosomal Survivin, a Plausible Biomarker for Early Detection of Prostate Cancer

**DOI:** 10.1371/journal.pone.0046737

**Published:** 2012-10-16

**Authors:** Salma Khan, Jessica M. S. Jutzy, Malyn May A. Valenzuela, David Turay, Jonathan R. Aspe, Arjun Ashok, Saied Mirshahidi, Dan Mercola, Michael B. Lilly, Nathan R. Wall

**Affiliations:** 1 Center for Health Disparities and Molecular Medicine, Department of Biochemistry and Microbiology, Loma Linda University, Loma Linda, California, United States of America; 2 Cancer Center and Department of Microbiology and Biospecimen Laboratory, Loma Linda University, Loma Linda, California, United States of America; 3 Division of Hematology/Oncology, Department of Medicine and Chao Family Comprehensive Cancer Center, University of California Irvine, Irvine, California, United States of America; 4 Department of Pathology and Chao Family Comprehensive Cancer Center, University of California Irvine, Irvine, California, United States of America; Sun Yat-sen University Medical School, China

## Abstract

**Background:**

Survivin is expressed in prostate cancer (PCa), and its downregulation sensitizes PCa cells to chemotherapeutic agents *in vitro* and *in vivo*. Small membrane-bound vesicles called exosomes, secreted from the endosomal membrane compartment, contain RNA and protein that they readily transport via exosome internalization into recipient cells. Recent progress has shown that tumor-derived exosomes play multiple roles in tumor growth and metastasis and may produce these functions via immune escape, tumor invasion and angiogenesis. Furthermore, exosome analysis may provide novel biomarkers to diagnose or monitor PCa treatment.

**Methods:**

Exosomes were purified from the plasma and serum from 39 PCa patients, 20 BPH patients, 8 prostate cancer recurrent and 16 healthy controls using ultracentrifugation and their quantities and qualities were quantified and visualized from both the plasma and the purified exosomes using ELISA and Western blotting, respectively.

**Results:**

Survivin was significantly increased in the tumor-derived samples, compared to those from BPH and controls with virtually no difference in the quantity of Survivin detected in exosomes collected from newly diagnosed patients exhibiting low (six) or high (nine) Gleason scores. Exosome Survivin levels were also higher in patients that had relapsed on chemotherapy compared to controls.

**Conclusions:**

These studies demonstrate that Survivin exists in plasma exosomes from both normal, BPH and PCa subjects. The relative amounts of exosomal Survivin in PCa plasma was significantly higher than in those with pre-inflammatory BPH and control plasma. This differential expression of exosomal Survivin was seen with both newly diagnosed and advanced PCa subjects with high or low-grade cancers. Analysis of plasma exosomal Survivin levels may offer a convenient tool for diagnosing or monitoring PCa and may, as it is elevated in low as well as high Gleason scored samples, be used for early detection.

## Introduction

Worldwide, prostate cancer (PCa) is the second most frequently diagnosed cancer and the sixth leading cause of cancer death in males [1], [2]. Increasing age, ethnicity and family history are the only established risk factors and there are no known preventable risk factors established to date [2]. Prostate cancers (PCa) are generally slow-growing malignancies that are characterized by an imbalance in the rates of cell division and cell death [3]. Surgery and radiation therapy are effective for localized disease but there is no effective treatment strategy for recurrent or metastatic PCa that has failed surgery, radiation or hormonal therapy [4]. An important challenge to develop treatments that are more effective depends upon our understanding of the molecular mechanism(s) of PCa progression, which will lead us to identify many potential therapeutic target genes and processes that are involved in apoptosis, cell proliferation, metastasis, and growth factor signaling. Total prostate-specific antigen (PSA) has revolutionized PCa screening and has resulted in an overall decrease in PCa metastasis and death [5]. Unfortunately, the application of PSA screening has also led to over-detection and overtreatment as PSA is neither cancer specific nor a surrogate for the biologic behavior of PCa [5], [6]. Elevations in PSA levels can reflect a cancer presence but can also be present as a result of infection, chronic inflammation or benign prostatic hyperplasia (BPH) [7], [8]. BPH has been shown to exist in greater than 70% of men over the age of 70 but is not considered to be a precursor of prostate cancer though they frequently coexist [9]. It is therefore necessary to continue to screen for biomarkers that are cancer-specific and that are detectable early in the course of the disease.

The processes of both cell survival and cell death have involved highly regulated signaling pathways that are currently the subject of intense investigation. It is known that regulation of apoptosis has a central role in the development of prostate cancer and its progression to an androgen-independent state, which is due, in part to up regulation of antiapoptotic genes after androgen deprivation [10]–[12]. Several lines of evidence suggest that one of the main events associated with progression after therapeutic failure is increased resistance to apoptosis [13], [14], mainly due to the up regulation of antiapoptotic genes, including Bcl-2, Bcl-X_L_, Mcl-1 [15], and Survivin [16]. Survivin, an inhibitor-of-apoptosis (IAP) protein family member, is associated with PCa development, progression, and drug resistance [17]–[20]. Recent evidence indicates that the overexpression of Survivin in PCa tumors is associated with poor prognosis and increased tumor recurrence [21]. In contrast, it has also been shown that knockdown of survivin expression by siRNAs enhances the chemosensitivity of prostate cancer cells, reducing tumorigenicity [22].

Traditionally, Survivin has been viewed as a cytoplasmic or nuclear protein. Recently, Survivin has been also shown to exist extracellularly, contained in small membrane bound vesicles known as exosomes [23], [24]. Exosomes are present in serum and urine and contain a wide range of proteins and RNAs and represent their tissue of origin making them a possible source or pool of novel PCa biomarkers [25]. Consistent with Survivin's association with unfavorable clinicopathological parameters, extracellular trafficking of Survivin throughout the tumor microenvironment could be responsible for augmenting the aggressive status of a tumor while prohibiting or minimizing therapeutic results. We have recently shown that exosome-bound Survivin protein can be secreted by cancer cells and be taken up by surrounding cells, producing a field effect that confers a general stress-survival phenotype.

Our present study was designed to investigate the existence of exosomal Survivin in the plasma of PCa patients with a variety of PCa presentations and to compare its exosomal expression levels to those found in control volunteers with no diagnosis of cancer and to patients diagnosed with benign prostatic hyperplasia or BPH. For the past 25 years the Gleason grading system has been used to help evaluate the prognosis of men with prostate cancer. Together with other parameters, prostate cancer has been staged as a means to predict prognosis and guide therapy [26]. Extracellular Survivin was found highly expressed in the plasma exosomes of PCa patients exhibiting Gleason scores of 6 (low) and 9 (high), and in patients who had relapsed on chemotherapy. However, there were no significant differences in Survivin levels between subjects with low or high Gleason scores. In addition, though exosomes containing Survivin were found in the serum from patients with a diagnosis of BPH, the overall level was significantly lower than that found in the plasma from PCa patients. We believe that in addition to diagnostic markers, prognostic, predictive and therapeutic markers are needed to act as surrogate endpoints in forecasting disease severity, choosing treatments, and monitoring responses to therapies [27]–[30]. Our demonstration of exosomal Survivin in the plasma of patient with newly diagnosed low-grade PCa provides a rationale for studies to investigate the utility of exosomal Survivin as an early, easily measured biomarker for PCa diagnosis. Exosomal Survivin may also be studied as a biomarker to monitor treatment of subjects with advanced PCa.

## Results

### Plasma levels of Survivin in healthy controls and PCa patients

**Figure 1:**
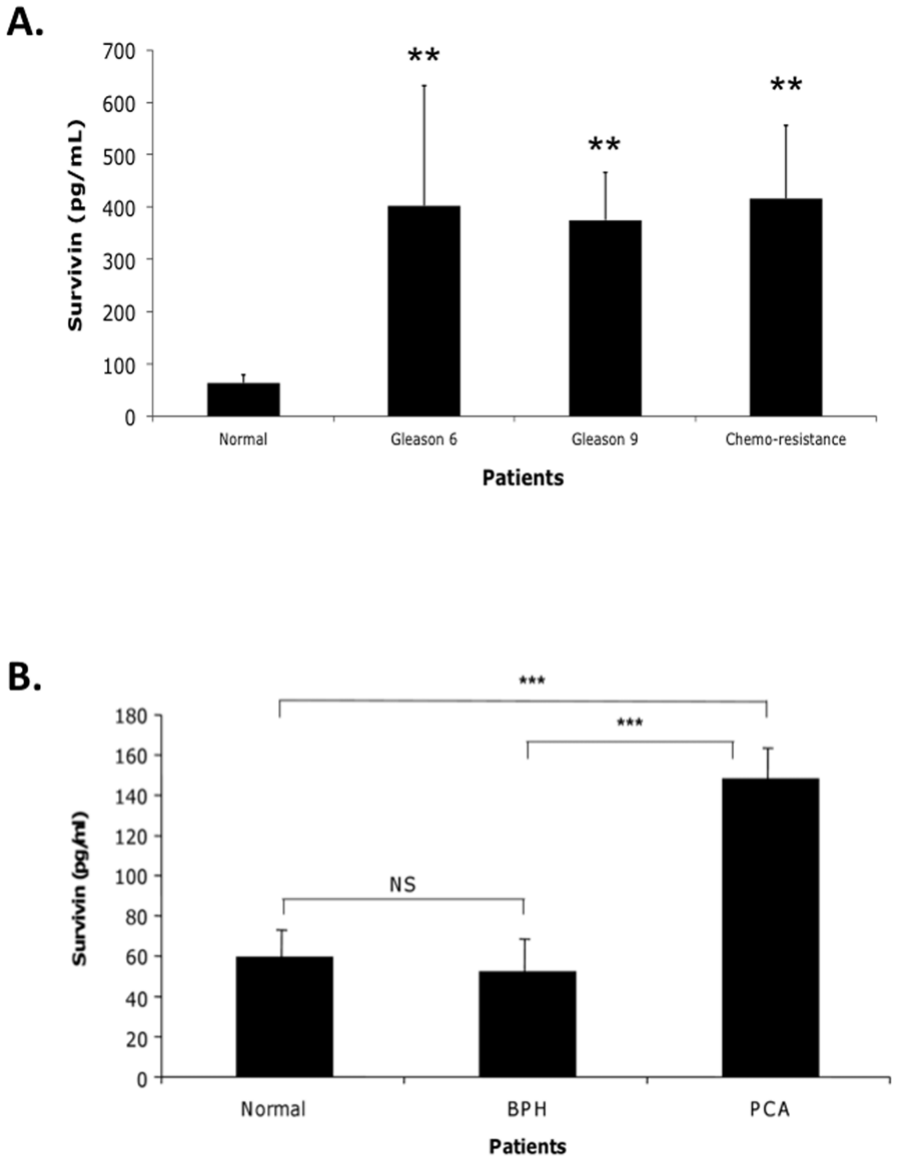
Quantification of Survivin levels in PCa plasma (A) and serum (B) samples by ELISA. **A**. Survivin levels were measured in plasma derived from Gleason 6 (n = 10), Gleason 9 (n = 10), and Taxotere-resistant subjects (n = 8). **B**. Survivin levels were measured in serum derived from BPH (n = 20), and PCa (n = 19). Comparisons were accomplished using MANOVA with normal healthy controls (n = 10 and 6 respectively). (**, p<0.05, ***, p<0.001; statistically significant).

Survivin was detectable in the plasma from all healthy control subjects and PCa patients. The measurement results of plasma Survivin in normal healthy controls and PCa patients are shown in **Figure 1A**. The mean plasma Survivin levels were significantly different between the healthy control subjects (61.5 pg/ml in controls [n = 10]) and the different cancer patient groups (Gleason 6 = 401.7 pg/ml [n = 10], Gleason 9 = 375.2 pg/ml [n = 10], and subjects resistant to the the chemotherapy agent Taxotere = 410 pg/ml [n = 8]; P<0.05 for each comparison vs. control). When the Survivin levels were compared among the three groups of PCa patients in two-way comparisons, none were significantly different from the others.

### Survivin can be collected from serum taken from healthy controls, BPH and PCa Patients

Like what has been measured above in plasma, Survivin was also detectable in the serum from patients having no prior diagnosis of cancer as well as in patients having the diagnosis of benign prostatic hyperplasia (BPH) and those diagnosed with prostate cancer (PCa) (**Figure 1B**). The mean serum Survivin levels were significantly (P<0.001) higher in the PCa subjects (150 pg/ml [n = 19]) than in the serum processed from normal controls (59.7 pg/ml [n = 6]) and from BPH patients (55 pg/ml [n = 21]).

### Plasma and Serum Survivin in PCa patients exists in an exosomal pool

**Figure 2:**
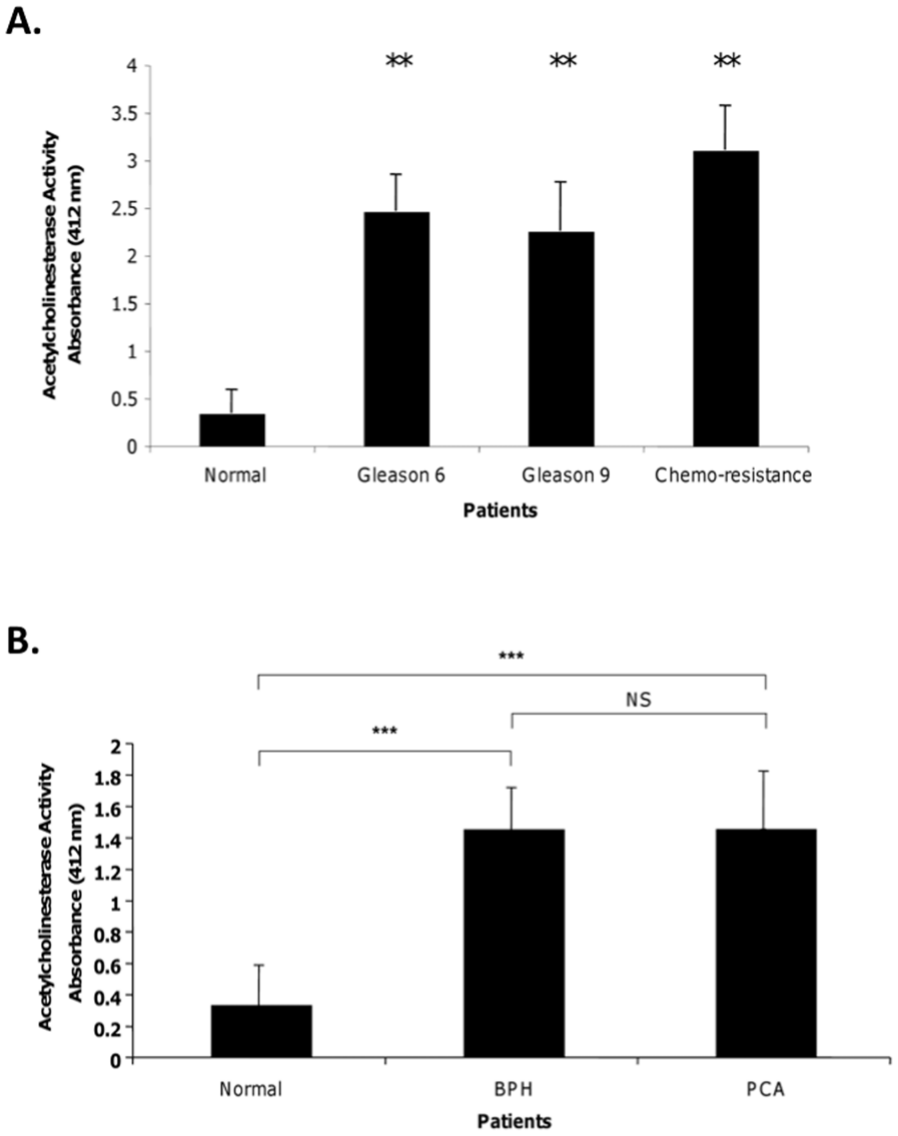
Exosomal contents in PCa patients plasma (A) and serum (B) samples by using the acetylcholinesterase activity assay. **A**. Exosome levels were measured in plasma derived from Gleason 6 (n = 10), Gleason 9 (n = 10), and Taxotere-resistant subjects (n = 8). **B**. Exosome levels were measured in serum derived from BPH (n = 20), and PCa (n = 19). Comparisons were accomplished using MANOVA with normal healthy controls (n = 10 and 6 respectively). (**, p<0.05, ***, p<0.001; statistically significant).

We have previously demonstrated that cultured PCa cells release Survivin into the extracellular milieu within exosomes [23]. These small, membrane-vesicles are also known to occur in the plasma as well as serum of cancer patients [31], [32]. Exosomes were therefore collected by differential centrifugation, and quantitated using the acetylcholinesterase enzymatic assays as we and others have previously described [24], [33]. The mean plasma exosome levels were significantly different between the healthy control subjects and the different cancer patients groups (P<0.001) whereas significant differences in exosome quantity were not found among the PCa patient sample groups (**Figure 2A**). This was also the case when comparing the exosome quantities found in BPH and PCa samples to that of the healthy control subjects (**Figure 2B**). Interestingly, there appears to be a measureable difference in Survivin and in exosomes depending upon the source as both Survivin and exosome quantities were higher when purified from plasma then when purified from serum.

### Plasma-derived exosomes contain Survivin

**Figure 3:**
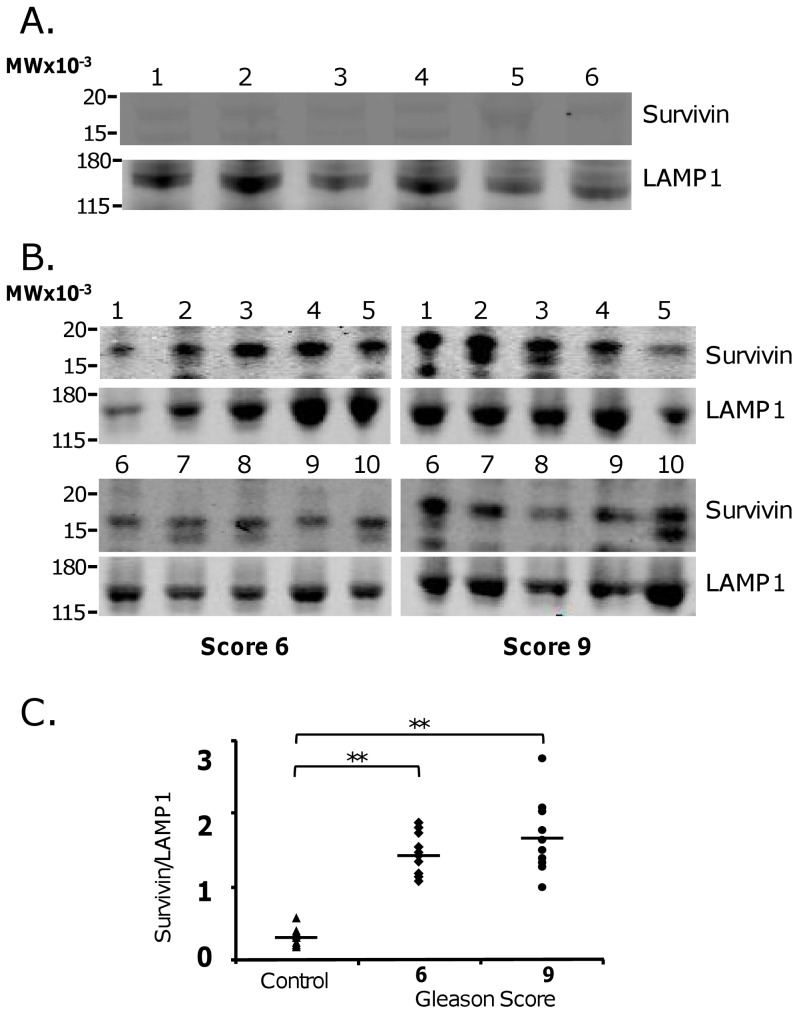
Western Blot Analysis of exosomal Survivin in untreated PCa plasmas. **A**. Antibodies for Survivin and Lamp1 were used for Western blotting of control patient-purified exosomal protein. **B**. Both Survivin and Lamp1 antibodies were detected in the Western Blotting of exosomes-derived from Gleason 6 and Gleason 9. **C**. Proportion analysis of Survivin density to Lamp1 density were shown in both Gleason 6, and Gleason 9 with normal healthy controls (**, p<0.05, statistically significant) with no significance recorded between Gleason 6 and Gleason 9 plasma-derived exosomes.

Exosomes were characterized by immunoblotting for the amount of Survivin protein and Lysosomal-associated membrane protein 1 (LAMP1). LAMP1 is a known exosome protein which is commonly used to ensure proper Western blot loading [24], [34], [35]. Exosomes isolated from PCa patient plasma exhibited enhanced Survivin loads compared to exosomes isolated from controls (**Figure 3**). Western blot analysis showed that little exosomal Survivin was detectable in plasma samples collected from six controls having no previous diagnosis of cancer in comparison to the exosome-specific protein Lamp1 (**Figure 3A**). In contrast, exosomes isolated from all twenty pre-treatment PCa subjects contained high amounts of Survivin protein compared to LAMP1 protein levels (**Figure 3B**). Interestingly, there was no significant difference in exosomal Survivin content between patients with Gleason 6 PCa and those with Gleason 9 PCa (**Figure 3C**) when normalized against Lamp1 (p<0.05).

### Differential expression of Survivin exists in BPH and PCa-derived exosomes

**Figure 4:**
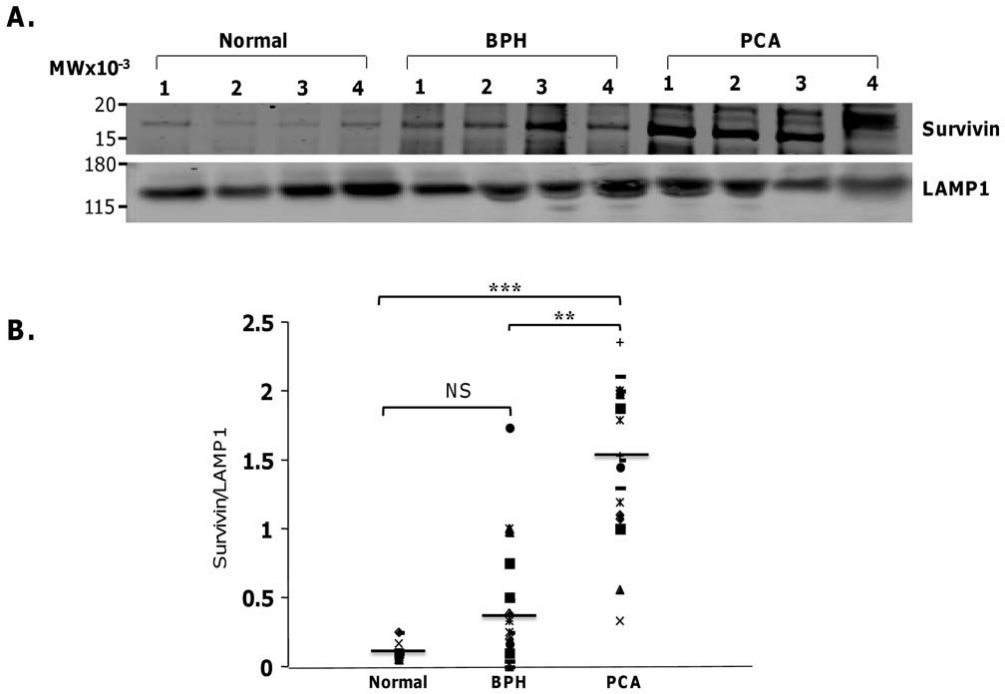
Western Blot Analysis of exosomal Survivin in normal control, BPH and untreated PCa serum samples. **A**. Antibodies for Survivin and Lamp1 were used for Western blotting of patient-purified exosomal protein. **B**. Proportion analysis of Survivin density to Lamp1 density were shown in both BPH and PCa with normal healthy controls (**, p<0.05, ***, p<0.001; statistically significant) with no significance recorded between normal controls and BPH.

As above, exosomes collected from BPH and PCa patient serums were characterized by immunoblotting for Survivin and LAMP1 protein (**Figure 4A**). Exosomes isolated from PCa and BPH patient serums exhibited enhanced Survivin loads compared to exosomes isolated from controls (**Figure 4A** **,** **Figure 4B** **and Figure S1**). Interestingly, though Survivin was detected in most and elevated in certain BPH patients, its overall level was significantly less than that found in PCa and there was no significant difference measured between BPH and control patient serums (**Figure 4B**).

### PCa patients with disease progression express high levels of exosomal Survivin

**Figure 5:**
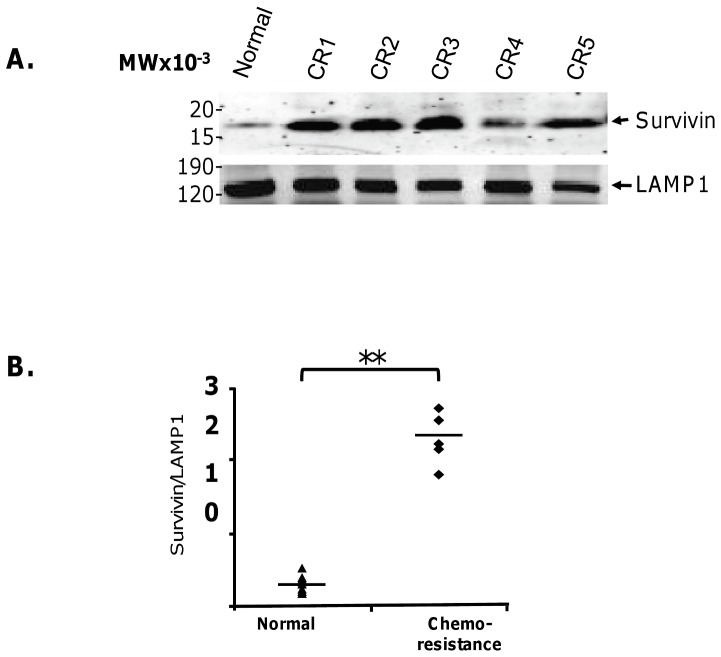
Western Blot Analysis of exosomal Survivin and Lamp1 in Taxotere-resistant PCa patients. **A**. Survivin and Lamp1 antibodies were shown positive. **B**. Densitometric analysis of Survivin/Lamp1 expression in a healthy control (normal) and chemoresistance (CR) cases (**, p<0.05, statistically significant).

Lastly, we evaluated exosomes collected from PCa patients who experienced disease progression while on treatment with chemotherapy. Like in our Gleason patient exosomes, Western blotting showed Survivin protein levels (**Figure 5A**) were markedly higher when compared to cancer-free control subjects, which was confirmed to be significant after densitometric analysis (**Figure 5B**).

### Clinical and pathological characteristics

Pretreatment data regarding initial plasma Survivin and PSA levels and Gleason scores were acquired for the patients studied. These pathological characteristics are detailed in **Tables 1** **,** **2** **,** **3** **,** **4**. In all, eight cases of prostate cancer recurrence after conventional treatment, ten cases of Gleason 6, ten cases of Gleason 9, (**Table 1**); and ten control cases (**Table 2**) were collected with plasmas analyzed. PSA from the plasma (clinical report) and from the ultracentrifuge-purified exosomes showed that though the protein concentrations were not identical, there was similarity in the trend of plasma to plasma-derived exosome PSA values. Importantly, Survivin amounts though significantly less in the exosomes were more consistent across the patients evaluated, and given the sensitivity of the ELISA, picograms for Survivin compared to nanograms for the PSA, is more accurate.

**Table 1:** Survivin ELISA and PSA levels of plasma from Chemo-resistance, Gleason 6, and Gleason 9 patients are shown.

Chemo-Resistance				
Patients ID#	Figure ID#	Survivin ELISA(pg/mL)	PSA* (ng/mL)	Exosomal PSA (ng/mL)
006	CR1	503+/−81.9	110	62.28+/−1.2
009	CR2	382+/−29.1	51.5	38.13+/−1.5
010	CR3	435+/−32.4	15.8	4.37+/−7
012	CR4	305+/−19.9	55.4	304.85+/−76.9
013	CR5	413+/−141.5	970	443.25+/−
002a	N/A	258+/−5.2	235	79.84+/−11.7
002b	N/A	246.6+/−183	8.9	1.32+/−0.4
007	N/A	327+/−10.8	62.9	31.91+/−6.6
**Gleason 6**				
028	1	332+/−15.3	3	2.43+/−0
044	2	328+/−54	2.4	2.90+/−0.2
061	3	330+/−34	5.8	0+/−1.1
066	4	332+/−4.5	8.1	6.10+/−0.5
077	5	313+/−26.8	4.4	5.63+/−0.7
085	6	1366.5+/−82.7	4	1.30+/−0.3
089	7	311+/−7.5	2.9	4.08+/−0.9
093	8	306+/−25.4	4.1	0.39+/−0
101	9	317+/−30.6	3.1	0.93+/−0
117	10	311+/−22.9	7	0+/−0.7
**Gleason 9**				
270	1	386+/−27.6	11	0.21+/−0.1
367	2	336+/−15.8	6.7	0.42+/−3.4
381	3	393+/−48.1	4.2	0+/−0.9
396	4	348+/−13.7	3.1	2.67+/−0
401	5	287+/−9.1	4.7	4.33+/−18
410	6	603+/−19.6	9.3	2.14+/−0
440	7	301+/−19.9	9.3	2.08+/−0.3
456	8	306+/−18.2	5.54	0.61+/−0.3
474	9	417+/−121.7	3.65.1	0.36+/−0.2
517	10	375+/−7.9	17.8	0+/−1.4

**N/A: Not available.**

* **Clinical PSA values.**

**Table 2:** Survivin ELISA and PSA levels of plasma from controls with no diagnosis of cancer.

Patients ID#	Figure ID#	Survivin (pg/mL)	Exosome PSA (ng/mL)
OPN 118	N1	67.5+/−0	0+/−0
OPN 119	N2	65+/−14.9	0+/−0.2
OPN 120	N3	75+/−7	0+/−0.3
OPN 121	N4	60+/−7	0+/−0.2
OPN 122	N5	45.75+/−1.4	0+/−1.0
OPN 123	N6	60+/−7.1	0+/−1.5
OPN 124	N7	67.5+/−0	0+/−.5
OPN 125	N8	71.6+/−10	0+/−1.5
OPN 126	N9	35+/−14	0+/−.5
OPN 127	N10	67.5+/−0	2.17+/−0.4

**Table 3:** Survivin ELISA and PSA levels of Sera from BPH patients are shown.

Patients ID#	Figure ID#	Survivin ELISA(pg/mL)	Exosomal PSA (ng/mL)
VCT08	1	44.5+/−4.94	0.955+/−
G918	2	46.5+/−0.7	0
4PHH	3	45+/−5.6	1.955
OSWG3	4	46+/−7	2.17
XHZN	5	64.5+/−6.36	0
87LUH	6	25+/−35.3	2.67
CC51Y	7	24.5+/−34.64	2.305
XHWCG	8	45.5+/−0.7	1.88
IOAYY	9	49.5+/−0.7	0
ZUQ1B	10	76.5+/−21.2	2.275
L8K8F	11	54.5+/−2.12	2.745
WBZVY	12	52.5+/−7.7	2.5
X3GDZ	13	49+/−1.14	1.955
9UXFW	14	89+/−2.8	0
JCO8M	15	55.5+/−2.12	2.35
XWKEL	16	53+/−11.3	0
H2GY6	17	45.5+/−4.9	2.035
PPAQH	18	54.5+/−12	0
AU4AD	19	66.5+/−3.5	0
DXCOB	20	70.5+/−0.7	0

**Table 4:** Survivin ELISA and PSA levels of Sera from Prostate Cancer patients.

Patients ID#	Figure ID#	Survivin ELISA(pg/mL)	PSA (ng/mL)*	Exosomal PSA (ng/mL)
642	1	140.1+/−0.34	6.2	0
6227	2	147.13+/−4.4	3.29	0
6636	3	154.21+/−7.24	4.5	0
3918	4	142.93+/−1.88	5.2	0
3960	5	140+/−0.03	13.5	0
2060	6	139+/−1.25	10.6	0
304	7	133.13+/−7	5.3	0
624	8	123.4+/−7.39	8.8	0
286	9	157.16+/−2.44	2.73	0
2828	10	155.75+/−5.7	3.9	0
14739	11	154.58+/−2.71	6.82	0
13077	12	155.7+/−2.1	7.1	1.73
6129	13	183+/−19.79	5.4	0
9508	14	179+/−26.87	9.4	1.835
14824	15	135.5+/−7	7	0
13406	16	146+/−2.82	8.8	2.335
9594	17	145+/−2.12	6.56	0
5117	18	149+/−7	42	0
69	19	146+/−5.6	58	0

* Clinical PSA Values.

Further studies using sera instead of plasma were performed on six controls, twenty BPH cases (**Table 3**) and 19 additional PCa cases (**Table 4**). Like before, ELISA results from Survivin provided a more sensitive and stable quantitation than did the ELISA for PSA. Quantitation from the sera of BPH patients showed an average of 52.9 pg/ml Survivin and 1.3 ng/ml PSA. In comparison, when evaluated from PCa patients, Survivin averaged 149 pg/ml and PSA averaged 0.3 ng/ml (**Tables 3** **and** **4**). BPH numbers for both Survivin and PSA are nearly 3 fold lower than in the PCa patient sera. Unfortunately, purchased BPH samples did not come with the clinical PSA ELISA quantities that we have from the PCa cases (**Table 3**).

### Correlations of Survivin with PSA

Of the patient's plasma/serum samples evaluated for Survivin, all samples (Gleason 6, Gleason 9, recurrence, or PCa samples) exhibited greater than 100 pg/mL Survivin (**Table 5**). In contrast, only 60% of Gleason 6, 80% of Gleason 9 plasmas and 84% of PCa sera had a PSA of greater than 4 ng/mL. Recurrent patient plasmas all had greater than 4 ng/mL. In control plasmas and BPH sera, no sample had a Survivin concentration greater than 100 pg/mL and no PSA concentration greater than 4 ng/mL (**Table 5**).

**Table 5:** Correlation of Survivin and PSA levels from Plasma.

Patients	Survivin	PSA
	(pg/mL)	(ng/mL)
**Normal**	<100 (n = 10) (**100%**)	<4 (n = 10) (**100%**)
	>100 (n = 0) (0%)	>4 (n = 0) (0%)
**Gleason 6**	<100 (n = 0) (0%)	<4 (n = 4) (40%)
	>100 (n = 10) (**100%**)	>4 (n = 6) (**60%**)
**Gleason 9**	<100 (n = 0) (0%)	<4 (n = 2) (20%)
	>100 (n = 10) (**100%**)	>4 (n = 8) (**80%**)
**Recurrences**	<100 (n = 0) (0%)	<4 (n = 0) (0%)
	>100 (n = 8) (**100%**)	>4 (n = 10) (**100%**)
**BPH**	<100 (n = 20) (**100%**)	<4 (n = 20) (**100%**)
	>100 (n = 0) (0%)	>4 (n = 0) (0%)
**PCA**	<100 (n = 0) (0%)	<4 (n = 3) (16%)
	>100 (n = 19) (**100%**)	>4 (n = 16) (**84%**)

## Discussion

Prostate carcinoma resists apoptosis with altered expression of both pro- and antiapoptotic proteins, thereby bypassing internal surveillance checkpoints, thriving in unfavorable microenvironments, and acquiring an invasive phenotype [20]. Recent studies suggest that inhibition of apoptosis, rather than enhanced cellular proliferation, is a critical trait that contributes to prostate carcinogenesis. Among the regulators of PCa apoptosis, interest recently has focused on Survivin, a multifunctional member of the inhibitor of apoptosis (IAP) gene family that counteracts cell death and controls mitotic progression. Selective overexpression of Survivin has been associated with higher tumor grade, advanced disease stage, rapid tumor progression, short patient survival, and resistance to therapy in patients with various malignancies [17]–[19].

Survivin exists in a number of subcellular locations such as the mitochondria, cytoplasm, and nucleus. Recently it has also been found in the extracellular space [23], [24]. We have shown that extracellular Survivin exists in multimolecular complexes which include heat shock proteins [24]. Furthermore, we have also shown that these complexes reside in or on exosomes, and that cancer treatment can enhance release of such exosomes from cancer cells [24]. Extracellular Survivin is able to mediate a pro-survival field effect through its secretion by cancer cells and uptake by surrounding normal and transformed cells [23]. Linking extracellular Survivin's ability to enhance cellular proliferation, survival and tumor cell invasion with a membrane-protective trafficking modality provides additional support for the hypothesis that Survivin plays a pivotal role in the pathobiology of cancer cell growth and protection from therapeutic interventions.

Several previous studies have examined the plasma levels of Survivin in cancer patients [36], [37]. In adult T-cell leukemia and the supernatants from in vitro cultures of solid-tumor cells, the levels of Survivin protein was low compared to the cellular Survivin protein and mRNA levels [37]. This finding resulted in the conclusion that Survivin protein levels in plasma do not reflect cellular Survivin levels. Furthermore, Survivin plasma levels were evaluated compared to alpha fetoprotein (AFP) in patients with chronic hepatitis C viral infection (HCV) with and without hepatocellular carcinoma (HCC) [36]. Though not concluded to be as reliable in these studies as AFP, Survivin levels were determined to be measurable and significant in patients suffering with HCV and measurable but not significant in those with HCC. Though not entirely clear, these results could be explained by the finding that HCV-infected HCC cells became more resistant to cell death and thus Survivin release compared to control or HCV-infected cells alone [36].

The prostate specific antigen (PSA) assay has been controversially utilized in prostate cancer screening though initially it was envisioned as a tool for evaluating treatment response [38]. Its use as a screening tool was driven by both the US prostate cancer burden and a need to detect its presence at a time that would allow for curative treatment to begin. Unfortunately, its use is believed to have led to both over diagnosis and overtreatment and hence the urgent need for novel biomarkers to be found to supplement PSA for management and treatment [39]. Exosomal Survivin measurements may provide another plasma-based assay for the presence of PCa. In the present study, we have identified for the first time that exosomes containing Survivin can be purified from plasma collected from patients with a diagnosis of PCa. Although there was little difference in exosome quantity, exosomal Survivin levels were higher in exosomes purified from PCa patients than from the sera of normal controls. In addition, in this study and that performed by others [36], [37], plasma-quantitated Survivin in samples taken from patients with no confirmed cancer diagnosis, also had reduced Survivin levels compared to PCa patient sera. The source of this exosomal Survivin pool, though unclear, may originate in immune cells [40] such as lymphocytes [41], monocytes [42] and dendritic cells [34], all Survivin containing cells [43] which have been shown to release exosomes. Survivin expression is associated with established features of biologically aggressive prostate carcinoma, such as higher final Gleason score and metastasis to regional lymph nodes [20]. In our study we found that patients who had failed treatment with Taxotere had elevated levels of exosomal Survivin. In addition, and of special interest, we found that patients presenting with either mid (Gleason 6) or high (Gleason 9) Gleason scores exhibited higher exosomal Survivin levels with no significant difference in Survivin content between them. Our findings, though more robust in the plasma we studied appear in agreement with what has been found with urine, tumor exosomes and PSA [31]. In these studies, urine exosome PSA is described as present in 20 of 24 PCa specimens while in our hands, tumor exosome Survivin is found in 47 of 47 specimens. Whether or not cancer treatment will affect tumor exosome Survivin levels has yet to be evaluated.

In this current study, we provide compelling evidence that circulating Survivin may be a useful diagnostic and prognostic marker for human PCa. Our results indicate that Survivin, analyzed directly from serum/plasma or from serum/plasma-derived exosomes, was lower in patients with BPH and healthy controls than in men with PCa. Survivin levels did not change, being consistently high, with regard to Gleason score and patients having recurrence, suggesting that Survivin levels could be used for early detection and could perhaps one day more accurately differentiate BPH from PCa. Comparing patients with and without tumors, but both having high PSA values will also be an important next step, as in our hands all the BPH samples we have acquired have relatively non-cancer PSA values [44].

The role of exosomal Survivin is still unknown. It is possible that a therapy-stressed, cellular release of exosomes containing Survivin and other antiapoptotic proteins, RNAs or miRNAs is performed as a final attempt to protect themselves from the stress that exists within the tumor microenvironment. Larger studies with more events and longer follow-up will be required to develop a more definitive statement regarding the association of Survivin expression in exosomes or in the tumor microenvironment with prostate carcinoma progression and as importantly, metastasis and survival. These findings will provide a rationale for further evaluation of exosomal Survivin and its role in resistance to androgen-based therapy in prostate carcinoma and raise the possibility of targeted combination therapy for advanced prostate cancer.

Our finding that Survivin, a unique human inhibitor of apoptosis (IAP), has intercellular transport and signaling capabilities is significant. Consistent with Survivin's association with unfavorable clinicopathological parameters, trafficking Survivin throughout the tumor microenvironment can drive the aggressive status of the tumor, prohibiting or minimizing therapeutic results. In our current work we show that though the overall number of exosomes being shed into patient plasma does not significantly change during the development of cancer, the level of Survivin in those exosomes increases significantly. Importantly, progression from mid- to late-stage does not drive an appreciable Survivin increase indicating that Survivin may prove useful as a biomarker for earlier detection of prostate cancer. Indeed, Survivin-based testing, performed on tumor-exosomes, will allow molecular-based diagnosis that in time may also aid in therapy decisions and disease response surveillance leading to better management of prostate cancer.

## Materials and Methods

### Patient Plasma and Serum

Plasma samples were collected from ten healthy male volunteers, and twenty-eight PCa patients. Plasma from twenty PCa subjects was obtained from the specimen bank of the SPECS consortium which is an observational clinical trial which utilizes prostate tissue and clinical values obtained by informed consent to derive gene signatures predictive of outcome at the time of diagnosis. SPECS is directed by Dr. Dan Mercola from the University of California, Irvine. Samples were pre-prostatectomy plasma randomly selected from ten low-grade PCa cases (Gleason 6) and ten high-grade PCa cases (Gleason 9). In addition, plasma from eight advanced-disease PCa patients participating in a second-line chemotherapy trial was also collected. These patients had failed chemotherapy with Taxotere. Nineteen serum samples were collected from PCa patients and six controls through the San Manuel Band of Mission Indians Biospecimen Laboratory at Loma Linda Universities Cancer Center. Twenty BPH samples were purchased from Bioserve Biotechnologies, Beltsville, MD. Bioserve Biotechnologies as provider of these samples is covered as defined in the HIPPA Act of 1996 as is providing them to us with a limited data set of protected health information.

Blood was collected in vacuum tubes containing sodium heparin. The tubes were centrifuged at 2000 g×7 minutes, and the plasma was then removed and aliquoted for storage at −80°C. All samples were obtained in the course of IRB-approved studies, following the documentation of informed consent in accordance with university policy at both Loma Linda University and the University of California at Irvine.

### Human Survivin Immunoassay

Whole plasma samples were subjected to a commercially available human Survivin Immunoassay (R&D systems, Minneapolis, MN) using the manufacturer's instructions in order to quantitate Survivin concentrations.

### Exosome Isolation

Plasma microvesicles were isolated as previously described, with minor modifications [45]. Thawed, cryopreserved plasma (2 ml) was centrifuged for 30 min at 500× g, 45 min at 12,000× g and 18 h at 110,000× g. Pellets were resuspended in a large volume of PBS, filtered through a 0.22-µm filter (Millipore, Billerica, MA) and centrifuged at 110,000× g for 1 h. Microvesicle pellets were washed once in a large volume of PBS, centrifuged at 110,000× g for 1 h and re-suspended in 50–200 ml of PBS. The amount of 110,000× g pellet proteins recovered was measured using the BCA protein assay kit (Pierce, Rockford, IL). Exosomes were used as fresh preparations for immunoblotting or were conserved at −80°C for later use. For serum samples, the commercially available ExoQuick (SBI, Mountain View, CA) was employed as described by the vendor. Briefly, 100 µL of serum was incubated with 100 µL of ExoQuick solution followed by a 2 hr incubation at 4°C followed by centrifugation at 1500× g for 30 minutes. After centrifugation the exosomes appear as a beige or white pellet at the bottom of the vessel which is then reconstituted with 500 µL of dH_2_O.

### Exosome quantification

To quantify the amount of exosomes released, we assessed the activity of acetylcholinesterase, an enzyme that is associated with these vesicles. Acetylcholinesterase activity was assessed as described by Savina et al. [46]. Briefly, 40 µl of the exosome fraction was suspended in 110 µl of PBS. 37.5 ml of this PBS-diluted exosome fraction was then added to individual wells on a 96-well flat-bottomed microplate. 1.25 mM acetylthiocholine and 0.1 mM 5,50-dithiobis(2-nitrobenzoic acid) were then added to exosome fractions in a final volume of 300 µl, and the change in absorbance at 412 nm was monitored every 5 min for 30 min.

### Human PSA Immunoassay

Isolated exosomes from patients' plasma were subjected to human Prostate Specific Antigen (PSA) using a PSA Immunoassy (American Research Products, Inc., Waltham, MA, USA) kit following the manufacturer's instructions in order to quantitate PSA concentrations in exosomes.

### Western Blot Analysis

For Western blot analysis, cells or exosomal preparations were lysed using lysis buffer (50 mM Tris (pH 7.5), 1% NP40, 0.25% DOC, 150 mM NaCl_2_, 1 mM PMSF, 10 µg/ml Aprotinin/leupeptin/pepstatin, 20 mM NaF, 0.2 mM EGTA, 1 mM EDTA (pH 8.0), H2O). For protein concentrations the BCA assay (Pierce, Rockford, IL) was used. Proteins from exosomes (20–40 µg) were separated using 12% Bis–Tris polyacrylamide gels, transferred onto polyvinylidene difluoride membranes (Millipore, Billerica, MA) and probed using the following antibodies: mouse monoclonal anti-LAMP1 (Abcam, Cambridge, MA), and rabbit polyclonal anti-Survivin (Novus, Littleton, CO). Secondary antibodies (IR-Dye conjugated) were goat anti-rabbit and goat anti-mouse immunoglobulin (LICOR, Lincoln, Nebraska). Immunoreactive bands were detected using the Odyssey Imaging System (LICOR, Lincoln, Nebraska) and quantified using ImageQuant software.

### Statistical Analysis

Multiple comparisons among different groups were calculated by using Multiple Analysis of Variance (MANOVA). Student t-test (two-tailed) was used to evaluate the significance of changes between control groups and experimental groups. Probability values P<0.05 were considered statistically significant.

## Supporting Information

Figure S1 **Western Blot Analysis of exosomal Survivin in normal control, BPH and untreated PCa serum samples.** Antibodies for Survivin and Lamp1 were used for Western blotting of patient-purified exosomal protein.
(TIF)
