# Supplementary figures and images for: Plasma-Derived Exosomal Survivin, a Plausible Biomarker for Early Detection of Prostate Cancer
Source: PLoS One. 2012 Oct 16;7(10):e46737. doi: 10.1371/journal.pone.0046737 (PMC3473028; doi:10.1371/journal.pone.0046737)

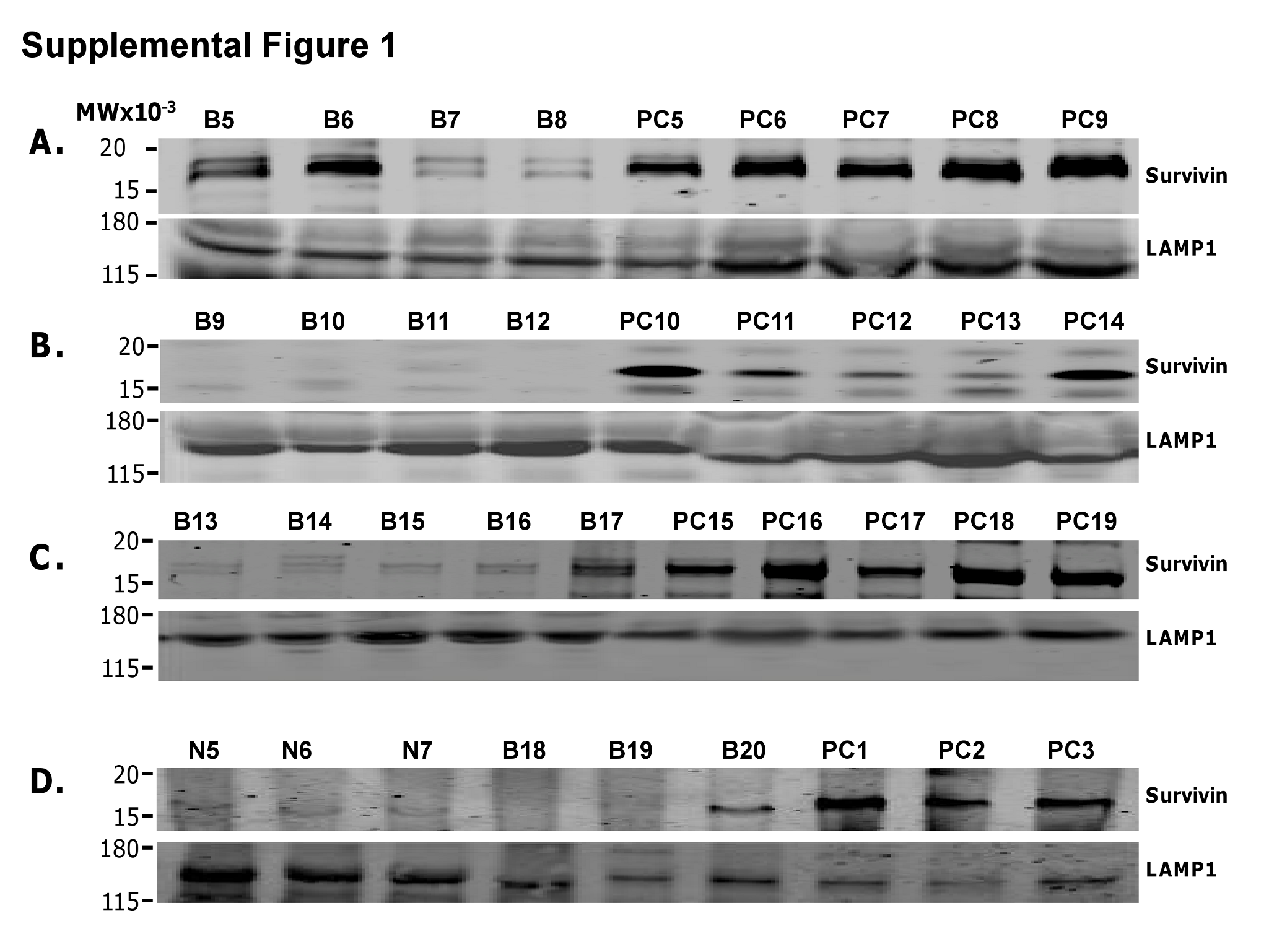

Supplement: Figure S1 — Western Blot Analysis of exosomal Survivin in normal control, BPH and untreated PCa serum samples. Antibodies for Survivin and Lamp1 were used for Western blotting of patient-purified exosomal protein. (TIF) [file pone.0046737.s001.tif]
